# Anti-Osteoarthritic Mechanisms of *Chrysanthemum zawadskii var. latilobum* in MIA-Induced Osteoarthritic Rats and Interleukin-1β-Induced SW1353 Human Chondrocytes

**DOI:** 10.3390/medicina56120685

**Published:** 2020-12-10

**Authors:** Jae-Hyuk Byun, Chi-Won Choi, Min-Jung Jang, Su Hwan Lim, Hae Jung Han, Se-Young Choung

**Affiliations:** 1 Department of Life and Nanopharmaceutical Sciences, Graduate School, Kyung Hee University, Seoul 02453, Korea; jaehyuk@khu.ac.kr; 2 Department of Preventive Pharmacy and Toxicology, College of Pharmacy, Kyung Hee University, Seoul 02453, Korea; ccwpharm@khu.ac.kr; 3 Department of Research, GREEN CROSS Wellbeing Co., Ltd., Seongnam 13595, Korea; mjjang@greencross.com (M.-J.J.); sswan32@greencross.com (S.H.L.); hjhan@gccorp.com (H.J.H.)

**Keywords:** *Chrysanthemum zawadskii var. latilobum*, osteoarthritis, MMPs, ADAMTS, SOX9

## Abstract

*Background and objectives: Chrysanthemum zawadskii* var. *latilobum* (CZ), which has traditionally been used as a oriental tea in Asia, is known to have anti-inflammatory effects in osteoarthritis (OA). But the mechanism of these effects has not been made clear and it needs to be elucidated specifically for the clinical use of CZE in OA. *Materials and Methods:* To reveal this mechanism, we first identified which biomarkers were expressed in the joints of rats in which OA had been induced with monosodium iodoacetate and determined whether CZ extract (CZE) could normalize these biomarkers in the progression of OA. The anti-osteoarthritis effect of CZE was evaluated for its capability to inhibit levels of extracellular matrix (ECM)-degrading enzymes and enhance ECM synthesis. We also sought to identify whether the marker compound of CZE, linarin, has anti-osteoarthritic effects in the human chondrosarcoma cell line SW1353. *Results:* The changes in matrix metalloproteinases (MMPs) were remarkable: among them, MMP-1, MMP-3, MMP-9 and MMP-13 were most strongly induced, whereas their expressions were inhibited by CZE dose dependently. The expressions of the ECM synthetic genes, *COL2A1* and *ACAN*, and the transcription factor SOX9 of these genes were reduced by OA induction and significantly normalized by CZE dose dependently. SOX9 is also a repressor of ECM-degrading aggrecanases, ADAMTS-4 and ADAMTS-5, and CZE significantly reduced the levels of these enzymes dose dependently. Similar results were obtained using the human chondrosarcoma cell line SW1353 with linarin, the biologically active compound of CZE. *Conclusions:* These anti-osteoarthritic effects suggest that CZE has mechanisms for activating ECM synthesis with SOX9 as well as inhibiting articular ECM-degrading enzymes.

## 1. Introduction

Osteoarthritis (OA) is a gradually degenerative disease of the joints characterized by the loss of articular cartilage. Although cartilage damage is the major symptom, the development of OA begins with biochemical changes due to the appearance of pro-inflammatory cytokines from the surrounding tissues of chondrocytes, such as the extracellular matrix (ECM), synovium or subchondral bone, as well as the chondrocytes themselves. Therefore, it is necessary to focus on these tissues and cells to inhibit OA progression. The ECM of articular cartilage consists of type 2 collagen and aggrecan. Chondrocytes synthesize the ECM and secrete proteolytic enzymes, leading to a balance of anabolic and catabolic action. Even in the presence of continued ECM synthesis by chondrocytes, the composition of collagen or aggrecan eventually degenerates if proteolytic enzyme levels increase, leading to the initiation of OA [1].

Therefore, the primary mechanism of OA progression is the excessive production of matrix metalloproteinases (MMPs), which degrade collagens in the ECM, and a disintegrin and metalloproteinase with thrombospondin motifs (ADAMTS) enzymes, which degrade aggrecans, whereas secondary causes include the inflammatory cytokines interleukin (IL)-1β and tumor necrosis factor (TNF)-α [2,3], prostaglandins, vascular endothelial growth factor (VEGF) [4] and reactive oxygen species such as nitric oxide (NO) [5].

In the clinical treatment of OA, nonsteroidal anti-inflammatory drugs (NSAIDs) are problematic for long-term use because of some side-effects.

Therefore, there is an urgent need for substances that block ECM-degrading enzymes, such as MMPs or ADAMTSs, by acting in the early stages of OA with few or no side effects [6], or substances that block the upstream factors associated with OA, such as inflammatory cytokines or nuclear factor-κB (Nf-κB) [7].

*Chrysanthemum zawadskii var latilobum* (CZ), which grows widely in Korea, Japan and China, is a perennial grass belonging to the family Asteraceae. It has been used as a tea in Asia for the purpose of protecting the liver and helping blood circulation, especially in gynecology. As an anti-inflammatory function of CZ, inhibition of IL-1β, TNF-α and NO production has also been confirmed and the mechanism has been elucidated [8]. The leaf extract of CZ inhibits NO production by inducing haem oxygenase-1 [9]. This anti-inflammatory function suggests the possible use of CZ for inhibiting OA progression. The first study to use an extract of CZ (CZE) in OA was conducted in 2016, and inhibition of IL-1, IL-6, TNF-α, MMP-1, MMP-3 and osteoclasts in rats with OA induced by type 2 collagen was confirmed [10]. In a second study, conducted in 2018, inhibition of MMP-2 and MMP-13 levels and inhibited apoptosis and enhanced autophagy in articular cartilage were observed following CZE treatment in rats with monosodium iodoacetate (MIA)-induced OA [11].

However, no study has compared inhibitory potency regarding MMPs associated with cartilage damage and confirmed ECM synthesis as well as inhibition of ADAMTSs by CZE. This study aimed to (1) determine which inflammatory and joint-related factors were changed in the MIA-induced rat model by CZE through microarray analysis; (2) compare the induction of six MMPs known to be associated with OA and the degree of inhibition by CZE; (3) identify the changes in the expressions of ADAMTS-4, ADAMTS-5, SOX9 (ADAMTS repressor) and some ECM synthesis-related genes transcribed by SOX9 in response to CZE; and (4) identify whether the marker compound of CZE, linarin, has anti-osteoarthritic effects in the human chondrosarcoma cell line SW1353 which is not a normal chondrocyte but has been established as a human chondrocyte model due to its representing the same arthritis-related biomarkers [12]. In particular, the comparison of MMPs and the identification of the effects of CZE on ADAMTS and SOX9 have not been revealed until now.

## 2. Materials and Methods

### 2.1. Preparation of CZE

CZE was prepared according to a previously published method [10]. The 70% ethanol extract of CZ (code name BST106) was supplied by the Green Cross WellBeing Corporation (GCWB, Seongnam-si, Korea). CZ was obtained from Jeongeup, Korea. Briefly, the active ingredient was extracted from dried whole plant *Chrysanthemum zawadskii var. latilobum* using 70% ethanol at 50 °C in a water bath and concentrated using a rotary evaporator at 50 °C. The concentrated extract showed a yield of roughly 20% with respect to the weight of raw material. The resulting CZE was then stored at room temperature. CZE was dissolved in dimethyl sulfoxide for the in vitro assays and suspended in 0.5% carboxymethyl cellulose for in vivo experiments.

### 2.2. High-Performance Liquid Chromatography Analysis of CZE

CZE was dissolved in distilled 50% ethanol and then analyzed using a high-performance liquid chromatography (HPLC) system (Agilent 1260, Santa Clara, CA, USA). CZE was separated using Phenomenex kintex column C18 (4.6mm × 250 mm, 5μm) at a flow rate of 1.0 mL/min. To detect chromatogram, the mobile phase was composed of 0.025M KH2PO4 (pH 3.0: phosphoric acid) in water (solution A) and 100% acetonitrile (solution B), gradient 0–12 min (15–25% B), 12-20 min (25–41% B), 20–30 min (41–90% B), and then equilibrated with 15% B for 5 min. Standard compounds for HPLC analysis were used: chlorogenic acid (Cat. No.: 0050-05-90, purity: 96.67%, HWI Group), isochlorogenic acid A (Cat. No.: BP0056, purity: 98.0%, Chengdu Biopurity Phytochemicals), isochlorogenic acid B (Cat. No.: BP0055, purity: 98.0%, Chengdu Biopurity Phytochemicals), isochlorogenic acid C (Cat. No.: CFN90354, purity: 98%, Chemfaces), linarin (Cat. No.: BP0115, purity: 97.63%, Chengdu Biopurity Phytochemicals).

### 2.3. Animals

Sprague Dawley rats weighing 200–220 g were purchased from RAON BIO Co. Ltd. (Yongin-si, Korea) and adapted for 1 week in the animal breeding room of the College of Pharmacy, Kyunghee University, under a 12-h/12-h light/dark cycle at a temperature of 21 °C ± 2 °C, a relative humidity of 50% ± 5% and a light intensity of 300–500 lux. After adaptation, only normal animals (as determined by observing the gross symptoms) were used for the experiment. The rats were allowed ad libitum access to common solid feed and water. The animal experiments were conducted with the approval of the Kyunghee University Laboratory Animal Ethics Committee (KHUASP(SE)-2018-026, 06.04.2018) and in accordance with the guidelines of the National Institutes of Health (NIH publication No. 86–23, revised 1985).

### 2.4. Induction of OA and the In Vivo Experimental Design

Following the method described by Hong et al. in 2018, 2 mg of MIA (Sigma-Aldrich, St. Louis, MO, USA) mixed with saline was injected into the joint cavity of each rat once. Since the effective dosage of CZE was previously determined to be 100 mg/kg, three doses of CZE (50, 100 and 200 mg/kg) were administered daily for 28 days. The number of rats per group was 12.

### 2.5. Staining for Histological Analysis

After all rats were sacrificed, knee articular tissue was obtained for histological analysis. The obtained knee articular samples were fixed in neutral-buffered formaldehyde. Then, the fixed samples were sliced into three paraffin-embedded sections (3 µm thick), followed by staining with hematoxylin and eosin (H&E) and safranin O. The cartilage thickness and proteoglycan area were measured using ImageJ software (National Institutes of Health, Bethesda, MD, USA).

### 2.6. Microarray Analysis

To identify the most effective biomarkers of OA, protein microarray analysis (RayBiotech, Norcross, GA, USA) was performed. Articular tissue was isolated from rats, treated with lysis buffer and centrifuged to extract its protein. Ninety proteins were analyzed via microarray performed in accordance with the manufacturer’s instructions. Briefly, the primary amines of the protein in articular cartilage tissue lysate were biotinylated. The biotin-labeled sample was added to the glass slide, which was preprinted with capture antibodies, and the slide was incubated to allow for interactions with the target proteins. Streptavidin-conjugated fluorescent dye (Cy3-equivalent) was then applied to the array. Finally, the glass slide was dried and laser scanning fluorescence microscopy (GenePix 4100A, Axon Instrument, Phoenix, AZ, USA) was used to visualize the signals. Data were analyzed using GenowizTM v4.0 (Ocimum Biosolutions, Hyderabad, India) software.

### 2.7. Human Chondrosarcoma Cell Line SW1353 Culture for In Vitro Experiment

For the in vitro test, human chondrosarcoma cell line SW1353 (No. HTB-94™) was purchased from the American Type Culture Collection (Manassas, VA, USA). SW1353 cells were cultured in Dulbecco’s modified Eagle’s medium F-12 (Welgene, Daegu, Korea) supplemented with 10% fetal bovine serum (Gibco BRL, Life Technologies Co., New York, NY, USA) and 1% penicillin/streptomycin at 37 °C in a 5% CO_2_ humidified atmosphere.

### 2.8. Induction of OA in Cell Culture and the In Vitro Experimental Design

To determine the proper concentrations of CZE, linarin (Biopurify Phytochemicals, Cheungdu, Sichuan, China) and indomethacin (Sigma-Aldrich, St. Louis, MO, USA) as positive controls, SW1353 cells were treated with six concentrations of CZE (5, 10, 20, 100, 200 and 500 μg/mL), linarin (0.5, 1, 5, 10, 50 and 100 μg/mL) and indomethacin (0.1, 1, 10, 20, 50 and 100 μM) to assess cytotoxicity and the assay was performed by using Cell Counting Kit-8 (Dojindo Molecular Technologies, Kumamoto, Japan) according to the manufacturer’s protocol. No cytotoxicity was observed for CZE concentrations ≤20 μg/mL, linarin concentration ≤0.5 μg/mL or indomethacin concentration ≤0.1 μM. Therefore the concentrations of CZE, linarin and indomethacin for the experiments using the human chondrocyte cell model were 20 μg/mL, 0.4 μg/mL (0.7 nM) and 0.1 μM respectively. After seeding 1 × 105 cells per well in 6-well plates and allowing cells to reach 80% confluency, the microenvironment of OA was induced using 10 ng/mL of IL-1β and cells were treated with CZE, linarin and indomethacin for 24 h.

### 2.9. RNA Isolation and Quantitative Real-Time Polymerase Chain Reaction 

Total RNA was extracted using easy-BLUE reagent (iNtRON, Seongnam, Korea). cDNAs were synthesized from RNA using a cDNA synthesis kit (TaKaRa, Tokyo, Japan). After cDNA synthesis, quantitative real-time polymerase chain reaction (qRT-PCR) was performed using SYBR Premix Ex Taq (TaKaRa) and an ABI StepOnePlus™ Real-Time PCR System (Applied Biosystems, Foster City, CA, USA). The reaction mixtures were incubated for an initial denaturation at 95 °C for 10 min, followed by 40 cycles of 95 °C for 5 s, 60 °C for 60 s and 72 °C for 30 s. The expression levels of the target genes were normalized to that of the reference β-actin gene. The primer sequences were as follows: aggrecan (ACAN), (forward) 5′-ATAACCCTGGCTCCT-3′ and (reverse) 5′-TCTGGACGTTAGCGG-3′; COL2A1, (forward) 5′-GAGCGGAGACTACTGGAT-3′ and (reverse) 5′-TCTGGACGTTAGCGG-3′; GAPDH (forward) 5′-CCTCGTCTC-3′ and (reverse) 5′-GGGTAG-3′.

### 2.10. Western Blot Assay

To obtain 30 μg of protein, articular cartilage was lysed in lysis buffer. The cells and tissue lysates were centrifuged at 13,000 rpm (16,609× *g*) for 15 min to collect the supernatants. The proteins were separated using sodium dodecyl sulfate-polyacrylamide gel electrophoresis and then transferred to polyvinylidene fluoride membranes. After incubation with primary antibodies, all bands were visualized using the appropriate horseradish peroxidase-conjugated secondary antibodies (Santa Cruz Biotechnology, GA, USA) and the ChemiDocTM XRS+ System (Bio-Rad, Richmond, CA, USA). Quantitative analysis was performed using Image LabTM software (Bio-Rad, HERCULES, Contra Costa County, CA, USA).

### 2.11. Statistical Analysis

All data, which were obtained from at least three independent experiments, were presented as the mean ± standard deviation. Statistical analysis was performed using one-way ANOVA followed by Tukey’s test in Statistical Package for the Social Sciences (SPSS, Chicago, IL, USA) software. Statistical significance was indicated by *p* < 0.05.

## 3. Results

### 3.1. HPLC Profile of CZE and Verification of Its Components

**Figure 1:**
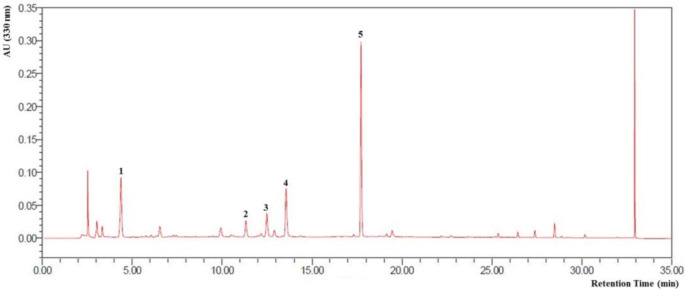
HPLC profile of CZE and its verified components. 1: Chlorogenic acid, 2: Isochlorogenic acid B, 3: Isochlorogenic acid A, 4: Isochlorogenic acid C, 5: Linarin.

The HPLC profile of CZE and its verified components are shown in Figure 1. The five peaks detected were identified as chlorogenic acid, isochlorogenic acid B, isochlorogenic acid A, isochlorogenic acid C and linarin in CZE. Linarin, the marker compound of CZE, is a natural flavonoid, which has been reported as a substance having antioxidant and anti-inflammatory activities. [13] These peaks were detected in the three batches produced from CZ. Furthermore, the batch-to-batch consistency of the three batches containing the marker compound, linarin, was shown to demonstrate a similarity value over 0.99 and its content was 22.8 mg/g.

### 3.2. CZE Reversed Weight Loss in MIA-Induced OA Rats

**Figure 2:**
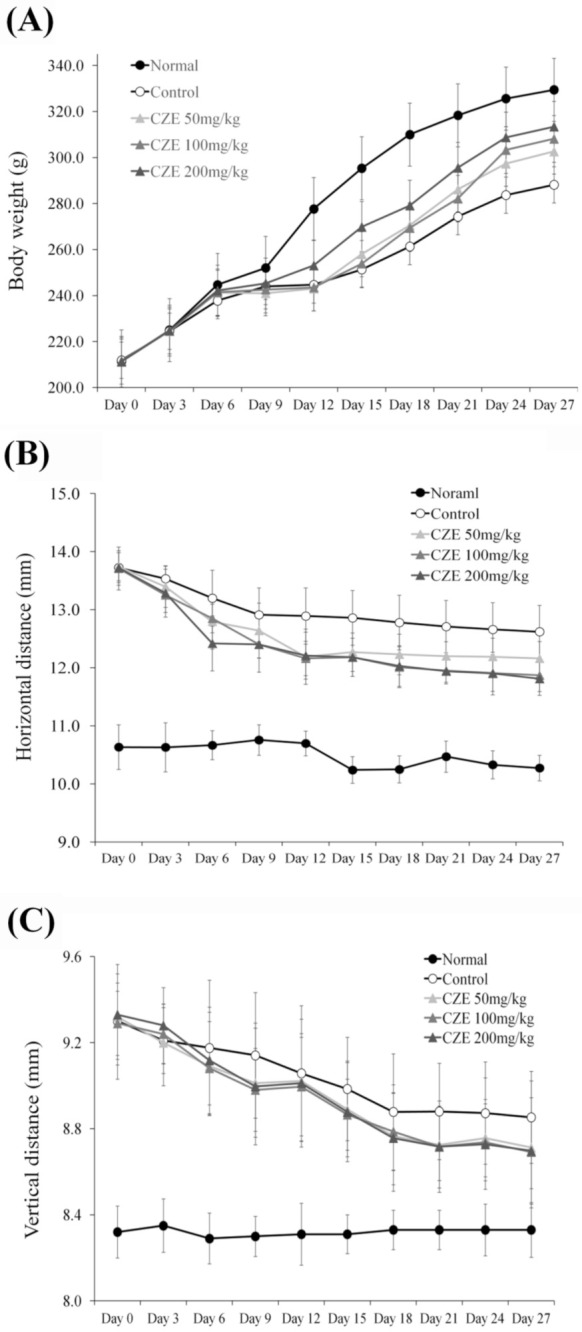
Changes in body weight as well as the horizontal and vertical sizes in edema of knee joints over four weeks in MIA-induced OA rats. (**A**) The body weight was measured every three days after MIA induction and from the 12th day the weight difference was shown for CZE treatment dose dependently. The horizontal (**B**) and vertical (**C**) lengths of articular edema from MIA induction were improved by CZE treatment. Data are presented as mean ± Standard Deviation (n = 6). Control, MIA induction; CZE 50 mg/kg, MIA induction + CZE 50 mg/kg; CZE 100 mg/kg, MIA induction + CZE 100 mg/kg; CZE 200 mg/kg, MIA induction + CZE 200 mg/kg.

Weight changes were investigated every three days after MIA induction and CZE treatment over a 28-day period. After 12 days, a weight difference was noted and eventually the weights of the rats decreased by 12.5% in the induction group relative to those in the normal group. Although the difference was not significant, the weight reduction was caused by decreases in movement and leg muscle mass because of MIA induction. Animal weights increased dose dependently in the 50, 100 and 200 mg/kg CZE groups, relative to those in the MIA-induction group (Figure 2A).

### 3.3. CZE Improved Articular Edema in MIA-Induced OA Rats

In the gross evaluation, calipers (M500-181-30, Mitutoyo, Japan) were used to measure the vertical and horizontal sizes of articular edema.

In the induction group, the vertical length increased relative to that in the normal group, but the length decreased with natural healing over time and remained constant longer than that of the normal group at 12 days. The vertical length was decreased by treatment with 50, 100 and 200 mg/kg CZE (Figure 2B). The horizontal length in the induction group increased relative to that of the normal group but the value was decreased by natural healing over time, although it remained 6% higher than that of the normal group on the final day. The horizontal length was reduced by treatment with 50, 100 and 200 mg/kg CZE versus that in the induction group (Figure 2C).

### 3.4. CZE Improved the Articular Structure in Mia-Induced OA Rats 

**Figure 3:**
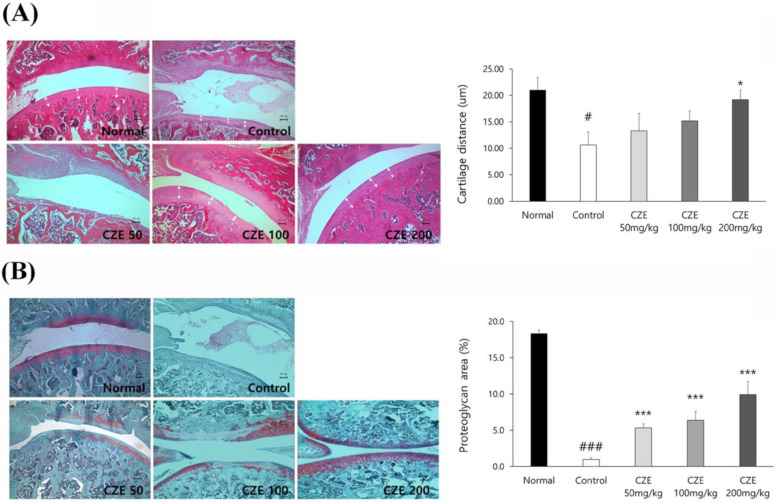
Effect of CZE on histological changes and quantitative data in the knee joints of MIA-induced OA rats. (**A**) The synovial tissue of cartilage was stained by hematoxylin and eosin (H&E) and the cartilage lengh (μm) was measured using ImageJ software. Scale bar (100 μm). (**B**) Aggrecan in cartilage was stained by safranin O and the aggrecan area (%) was measured using ImageJ software. Data are presented as mean ± SEM (n = 6). Control, MIA induction; CZE 50 mg/kg, MIA induction + CZE 50 mg/kg; CZE 100 mg/kg, MIA induction + CZE 100 mg/kg; CZE 200 mg/kg, MIA induction + CZE 200 mg/kg. Significantly different from the normal or control value: # *p* < 0.05, ### *p* < 0.001 vs. normal; * *p* < 0.05, *** *p* < 0.001 vs. vontrol.

The H&E staining revealed that the synovial tissues of the cartilage and capsule were maintained in the normal group, whereas the synovial membrane was damaged and cartilage layer thickness was decreased in the induction group. In the CZE treatment groups, less synovial membrane damage was observed and the condition and thickness of the cartilage tissue were recovered relative to the findings in the induction group. The cartilage length was decreased in the induction group relative to that in the normal group, whereas the length was increased by treatment with 50, 100 and 200 mg/kg CZE compared with that in the induction group (Figure 3A).

Reddish staining of aggrecan was observed upon safranin O staining, indicating that the joints were normal. In the induction group, MIA caused severe cartilage erosion around the joints and the red color of aggrecan almost disappeared. In the CZE treatment groups, safranin O staining illustrated that the extent of aggrecan erosion was low. The area of aggrecan staining was reduced in the induction group relative to that in the control group. Meanwhile, compared with the findings in the induction group, the area of aggrecan staining was increased by treatment with 50, 100 and 200 mg/kg CZE (Figure 3B).

### 3.5. Pro-Inflammatory and ECM-Degrading Marker Levels Were Increased in the Articular Cartilage of MIA-Induced OA Rats

Since this study aimed to evaluate the efficacy of CZE in improving OA symptoms, microarray analysis was performed prior to the full-fledged study to identify OA-related factors and changes in their expression induced by CZE (100 mg/kg) in order to select efficient biomarkers. Among 90 factors, 34 exhibited meaningful changes in expression, which could be divided into three groups: (1) pro-inflammatory factors, (2) MMPs, (3) other factors (Table 1). Many inflammatory factors in group 1 were upregulated in the induction (control) group relative to those in the normal group levels, and this overexpression was reversed in the 100 mg/kg CZE group. In addition, the expression of growth factors such as VEGF was increased by OA induction but inhibited by CZE. Among other large changes, the expressions of ubiquitin and adiponectin were decreased by OA induction but increased by CZE treatment. The changes in MMP-13 levels were more remarkable than those of the other factors. OA induction increased MMP-13 expression by 41%, whereas CZE (100 mg/kg) significantly decreased MMP-13 levels by 48% compared with the levels in the induction group. Since only MMP-2, MMP-8 and MMP-13 were identified via microarray, MMP-1, MMP-3 and MMP-9, which were missing among OA-related MMPs, were further examined using Western blotting. Since these three MMPs exhibited tendencies similar to those of MMP-13 in the microarray, MMPs are extremely influential to development of OA.

### 3.6. CZE Decreased the Levels of Six MMPs in Articular Cartilage of MIA-Induced OA Rats

After the six MMPs were identified via microarray and Western blotting, Western blotting revealed dose-dependent inhibition of articular degeneration in the CZE groups.

In the induction group, MMP-13, MMP-1, MMP-9, MMP-3, MMP-2 and MMP-8 expressions were increased by 126.5%, 81.6%, 76.3%, 66.6%, 37.4% and 30.8%, respectively, relative to the control levels.

**Figure 4:**
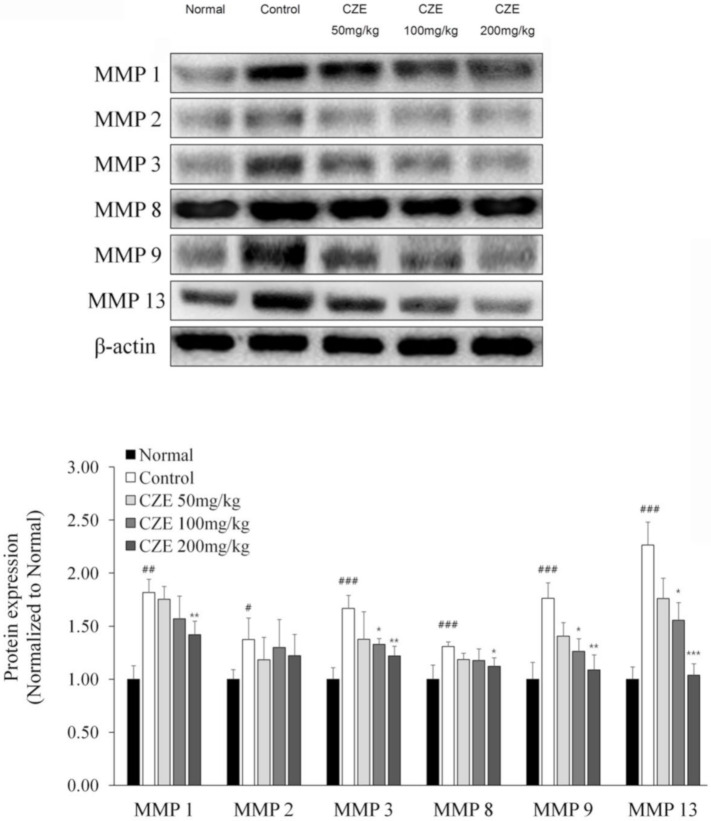
Inhibitory effect of CZE (50, 100 and 200 mg/kg) on six MMPs in MIA-induced OA rats. The expressions of six MMPs in the knee joints were compared. Data are presented as mean ± SEM (n = 6). Control, MIA induction; CZE 50 mg/kg, MIA induction + CZE 50 mg/kg; CZE 100 mg/kg, MIA induction + CZE 100 mg/kg; CZE 200 mg/kg, MIA induction + CZE 200 mg/kg. Significantly different from the normal or control value: # *p* < 0.05, ## *p* < 0.01, ### *p* < 0.001 vs. normal; * *p* < 0.05, ** *p* < 0.01, *** *p* < 0.001 vs. control.

MMP-13 and MMP-9 levels were significantly decreased in a dose-dependent manner in the CZE groups. In addition, MMP-1 and MMP-3 levels were also decreased dose dependently. Meanwhile, no dose-dependent effects were observed on MMP-2 and MMP-8 expression (Figure 4).

### 3.7. CZE Increased the Expression of SOX9 and ECM Synthetic Genes in Articular Cartilage of MIA-Induced OA Rats

**Figure 5:**
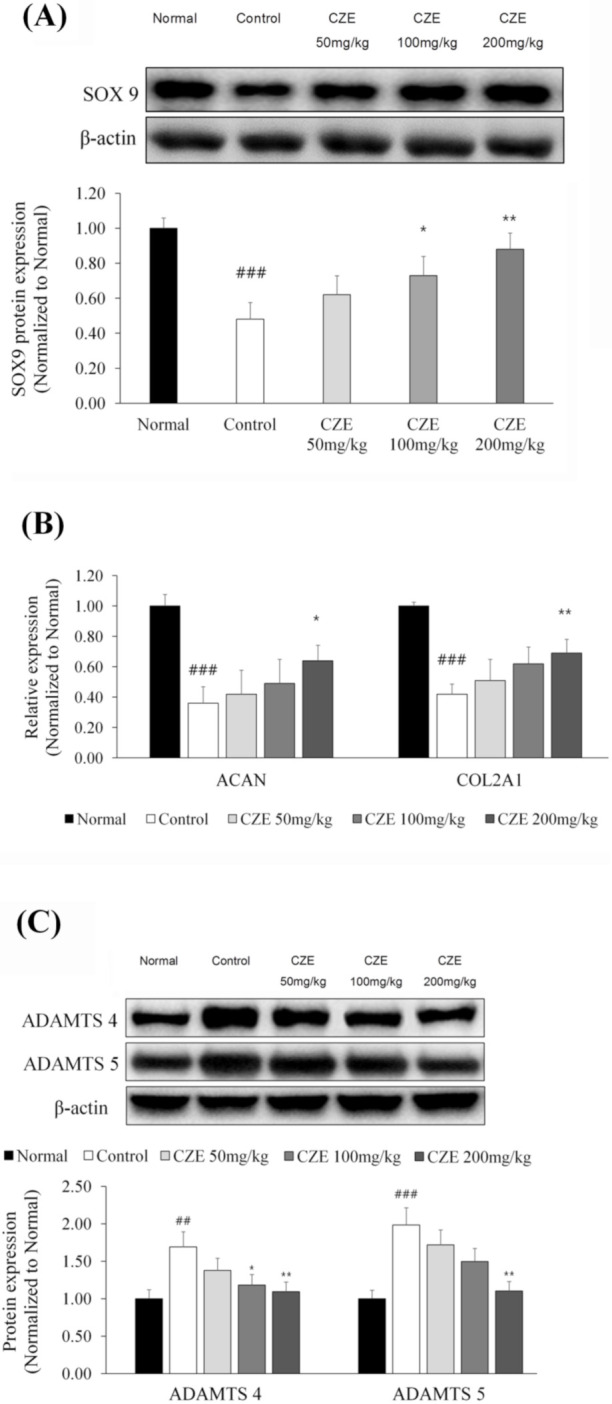
Effect of CZE (50, 100 and 200 mg/kg) on ECM synthetic factors and inhibitory effect of CZE on two ADAMTSs in the knee joints of MIA-induced OA rats. The expressions of SOX9 (**A**) and the ECM synthesis-related genes, *ACAN* and *COL2A1* (**B**), were identified. The expressions of ADAMTS-4 and ADAMTS-5 (**C**) in the knee joints were compared. Data are presented as mean ± SEM (n = 6). Control, MIA induction; CZE 50 mg/kg, MIA induction + CZE 50 mg/kg; CZE 100 mg/kg, MIA induction + CZE 100 mg/kg; CZE 200 mg/kg, MIA induction + CZE 200 mg/kg. Significantly different from the normal or control value: # *p* < 0.05, ## *p* < 0.01, ### *p* < 0.001 vs. normal; * *p* < 0.05, ** *p* < 0.01, *** *p* < 0.001 vs. control.

SOX9 has a protective role as a repressor of aggrecanases, ADAMTS-4 and ADAMTS-5 and acts as a transcription factor for the ECM-producing genes *ACAN* and *COL2A1* [14]. SOX9 expression was reduced by 52% in the induction group relative to that in the normal group, whereas this downregulation was reversed dose dependently in the 50, 100 and 200 mg/kg CZE groups (Figure 5A).

The gene expressions of *ACAN* and *COL2A1*, which were upregulated by SOX9, were evaluated. *ACAN* expression was reduced by 64% in the induction group relative to the normal group level. Compared with the level in the induction group, *ACAN* expression was significantly increased dose dependently in the 50, 100 and 200 mg/kg CZE groups. *COL2A1* expression was reduced by 58% in the induction group compared with that in the normal group, Relative to the induction group level, *COL2A1* expression was significantly increased dosedependently in the 50, 100 and 200 mg/kg CZE groups (Figure 5B).

### 3.8. CZE Decreased the Levels of Two ADAMTSs in Articular Cartilage of MIA-Induced OA Rats

To identify the effect of CZE on aggrecan, the levels of ADAMTS-4 and ADAMTS-5 were evaluated. These levels were increased by 69 and 98%, respectively, in the induction group. Meanwhile, compared with the induction group levels, ADAMTS-4 levels were decreased dose dependently in the 50, 100 and 200 mg/kg CZE groups and ADAMTS-5 levels in these groups were also lowered dose dependently (Figure 5C).

### 3.9. CZE Decreased Serum IL-1β and TNF-α Levels in MIA-Induced OA Rats

**Figure 6:**
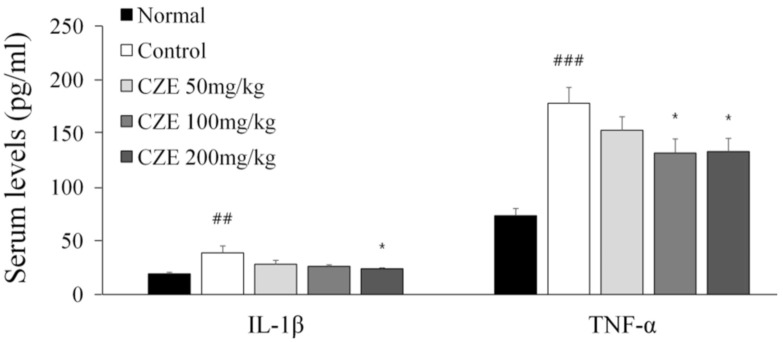
Inhibitory effect of CZE (50, 100 and 200 mg/kg) on levels of serum IL-1β and TNF-α in MIA-induced OA rats. Data are presented as mean ± SEM (n = 6). Control, MIA induction; CZE 50 mg/kg, MIA induction + CZE 50 mg/kg; CZE 100 mg/kg, MIA induction + CZE 100 mg/kg; CZE 200 mg/kg, MIA induction + CZE 200 mg/kg. Significantly different from the normal or control value: ## *p* < 0.01, ### *p* < 0.001 vs. normal; * *p* < 0.05 vs. control.

We confirmed that CZE could reserve changes in serum IL-1β and TNF-α levels induced by MIA. IL-1β was increased by 102.6% in the induction group relative to the normal group level. IL-1β was significantly decreased dose dependently in the 50, 100 and 200 mg/kg CZE groups, compared with the induction group level. TNF-α was increased by 142.4% in the induction group relative to that in the normal group. Compared with the induction group level, TNF-α was significantly decreased dose dependently by 50, 100 and 200 mg/kg CZE (Figure 6).

### 3.10. Linarin, the Marker Compound of CZE, Decreased the Levels of MMPs in IL-1β-Treated Human Chondrosarcoma Cell Line SW1353

Through in vivo experiments using rats, ECM-degrading enzymes, such as MMPs, were found to be major biomarkers of OA and the inhibitory effect of CZE was confirmed. Since ECM-degrading enzymes are mainly produced in chondrocytes among joint-constituting cells [15,16], human chondrocytes, which are the key cells involved in OA progression, were cultured and identified to confirm whether the same results could be obtained for the same biomarkers.

For in vitro experiments, it is most suitable to use isolated normal chondrocytes from the human cartilage, but as it is actually very difficult to acquire these, the human chondrosarcoma cell line SW1353 has been established as a human chondrocyte model. Therefore, the human chondrosarcoma cell line SW1353 was chosen to identify the efficacy of CZE and linarin. Since HPLC component analysis confirmed that the marker compound was linarin, we identified whether linarin had an anti-osteoarthritic effect.

Through the cytotoxicity assay, the concentrations of CZE and its marker compound linarin were determined. The concentration of linarin was determined by the amount of its percentage in CZE.

**Figure 7:**
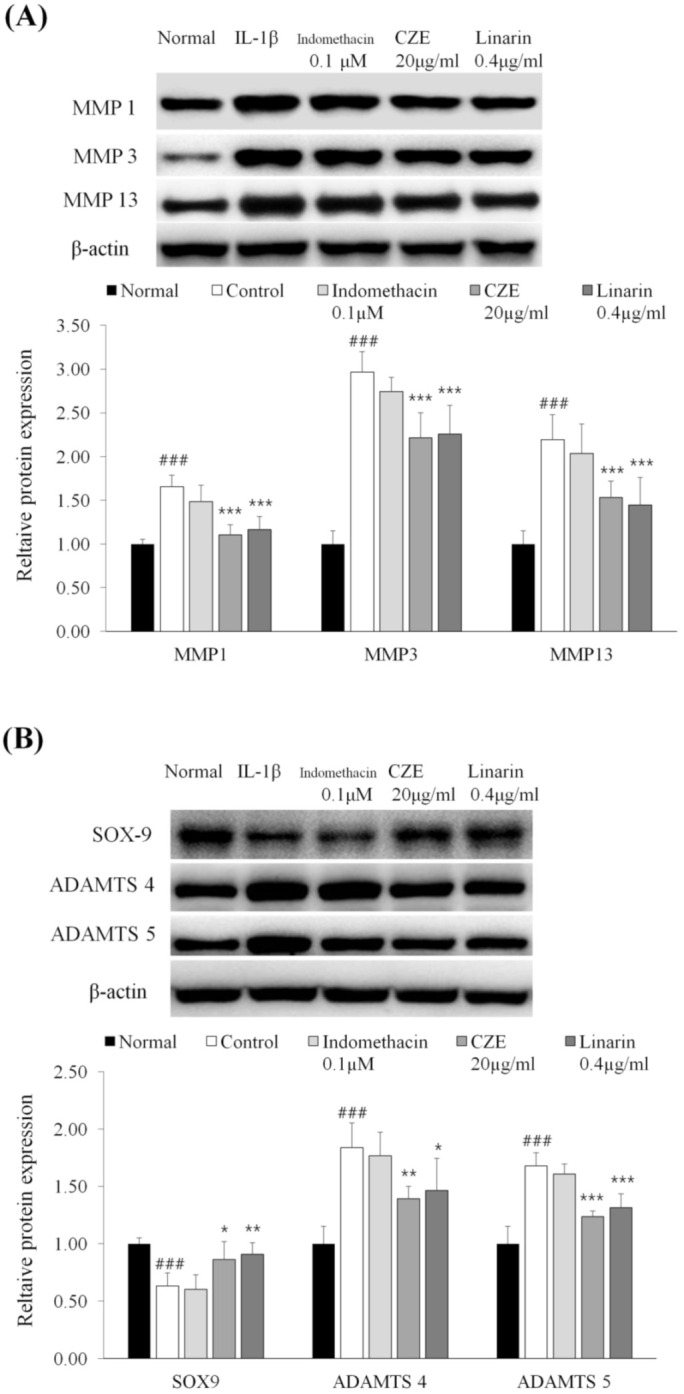
Inhibitory effect of indomethacin (0.1 μM), CZE (20 μg/mL) and linarin (0.4 μg/mL) on three MMPs (**A**), SOX9 and two ADAMTSs (**B**) in human chondrosarcoma cell line SW1353 treated with interleukin-1β. Data are presented as mean ± SEM (n = 6). Control, interleukin-1β treatment; indomethacin 0.1 μM, interleukin-1β treatment + indomethacin 0.1 μM; CZE 20 μg/mL, interleukin-1β treatment + CZE 20 μg/mL; linarin 0.4 μg/mL, interleukin-1β treatment + linarin 0.4 μg/mL. Significantly different from the normal or control value: ### *p* < 0.001 vs. normal; * *p* < 0.05, ** *p* < 0.01 and *** *p* < 0.001 vs. control.

MMP-1, 3 and 13, which were confirmed as the main ECM-degrading enzymes in in vivo experiments, were investigated and similar findings were recorded. The increases in IL-1β-treated human chondrosarcoma cell line SW1353 were 65.6% for MMP-1, 196.5% for MMP-3 and 119.3% for MMP-13. Relative to the levels for IL-1β-treated human chondrosarcoma cell line SW1353, the expressions of MMP-1, MMP-3 and MMP-13 were decreased dose dependently in the 20 μg/mL CZE and 0.4 μg/mL linarin treatment (Figure 7A).

Since 0.4 μg/ml of linarin exhibited inhibitory effects equivalent to MMPs, linarin can be regarded as the main biologically active compound of CZE.

### 3.11. Linarin Increased the Expression of SOX9 and Decreased the Levels of Two ADAMTSs in IL-1β-Treated Human Chondrosarcoma Cell Line SW1353

SOX9 expression was reduced by 36.6% in IL-1β-treated human chondrosarcoma cell line SW1353 and this downregulation was reversed dose dependently in the 20 μg/mL CZE and 0.4 μg/mL linarin treatments.

ADAMTS-4 and ADAMTS-5 levels were increased by 83.7 and 82.5%, respectively, in IL-1β-treated human chondrosarcoma cell line SW1353. Compared with IL-1β-treated human chondrosarcoma cell line SW1353, ADAMTS-4 and ADAMTS-5 expression were decreased dose dependently in the 20 μg/mL CZE and 0.4 μg/mL linarin treatments (Figure 7B). These results showed the same tendency as the MMPs results above.

## 4. Discussion

The pathological progression of OA is complex, but in the early stages the condition shares characteristics with other inflammatory diseases. In other words, when external stimuli such as load compression stimulate cartilage cells, IL-1β and TNF-α are produced to activate transcription factors including Nf-κB, which upregulates secondary inflammatory cytokines, such as IL-6, IL-8, IL-12, prostaglandin E_2_ and NO. These induce the production of MMPs and ADAMTSs, which eventually destroy articular cartilage. To prevent the progression of OA, inhibitors of early-stage factors, such as IL-1β and TNF-α, are used in clinical therapy, but it is important to identify more selective biomarkers that only target the joints. To determine how efficiently CZE can inhibit OA progression, this study first looked for biomarkers associated with OA progression using microarray analysis. In addition to IL-1β and TNF-α, various inflammatory factors were upregulated by OA induction and inhibited by CZE, but the change in MMP-13 was the most significant. An increase in VEGF due to OA induction was identified. Increased expression of VEGF is associated with severe progression of OA [17]. In addition to VEGF, its associated factors, such as epidermal growth factor receptor and neurofilin-2, were increased and CZE inhibited them. In addition, the expression of ubiquitin, which helps break down and clean up aging proteins, was increased by CZE significantly. Ubiquitin upregulation has been implicated in a variety of diseases via its promotion of autophagy [18] and the association between OA and autophagy has been well studied [19,20]. Therefore, the upregulation of ubiquitin by CZE strongly supports the clinical use of the substance in OA. Additionally, the increased expression of adiponectin in joint tissue induced by CZE may also be beneficial for relieving OA. In particular, adiponectin levels in synovial fluid tend to decrease during OA progression, whereas adiponectin exerts a protective effect in chondrocytes because it increases tissue inhibitor of metalloproteinase 2 expression and lowers IL-1β-induced MMP-13 expression [21]. Another study found that adiponectin concentrations in the subchondral bone of human lumbar facet joints decrease during OA progression [22]. These studies found that adiponectin levels in articular cartilage tissue decreased during OA progression, whereas other studies observed increased adiponectin levels in serum but not joint tissue [23,24]. Therefore, the relationship between adiponectin levels in joint tissue and serum is unknown.

MMPs can be divided into collagenases (MMP-1, MMP-8 and MMP-13), gelatinases (MMP-2 and MMP-9) and stromelysins (MMP-3 and MMP-10) [25]. The gelatinases degrade gelatin, which is a collagen fragment created by collagenase, and various types of collagens and structures connecting ECM and cartilage cells, such as fibronectin, elastin and laminin [26]. Studies have shown that among MMPs, MMP-13 is a main biomarker related to OA [27,28], in line with our findings. However, because there has been no comparative study between MMPs, the MMPs that could serve as biomarkers of OA have not been identified. In the rat model, the collagenase MMP-1, MMP-3, MMP-9 and MMP-13 levels were elevated highly and showed significant dose dependent decreases due to CZE. This was the first study to compare which MMPs had a significant impact on OA progression and identify the effectiveness of CZE. The present finding that MMP-2 was less responsive to treatment than MMP-1 and MMP-13 in both models could be attributable to the fact that gelatinase is more active in subchondral bone than in ECM [29].

A prior meta-analysis found that MMP-3 expression appeared to be higher in synovial fluid than in serum [30]. In another meta-analysis involving 753 patients, MMP-1 expression was higher in synovial fluid than in serum, but that of the gelatinases MMP-2 and MMP-9 was higher in serum [31]. This supports the results of this present study, which showed low induction of MMP-2 and its non-CZE dose-dependent inhibition. In addition, this same meta-analysis also found differences in the prevalence of MMPs among Asian and Caucasian populations. Higher levels of MMP-1 and MMP-2 were found in Asians than in Caucasians, suggesting that CZE might be more effective in Asian populations. Recently, single-nucleotide polymorphisms related to diseases have been studied. Polymorphism of MMPs has been well studied in cancer, coronary artery disease and glaucoma [32]. Since MMP expression is also a major cause of OA, the results of this study can be applied to the clinical treatment of OA. In other words, because MMP-1, MMP-3, MMP-9 and MMP-13 were the most efficient biomarkers among the tested MMPs and the significant inhibition of MMP expression by CZE was verified, CZE can be used to treat specific individuals in whom MMP-1, MMP-3, MMP-9 and MMP-13 are highly expressed because of polymorphism.

Aggrecan has important roles in maintaining healthy joints. Aggrecan has a structure consisting of glycosaminoglycan (GAG) attached to the core protein branch, which is rejoined to the hyaluronic acid stem to fill the ECM space. Therefore, to maintain soft joints, an abundance of aggrecan and collagen is extremely important [33]. Blocking of ADAMTSscan prevent the destruction of the core protein branch of aggrecan, which is important for relieving OA [34]. Among the ADAMTSs, ADAMTS-4 and ADAMTS-5 are well known as the main causes of joint damage [35,36,37]. A previous study reported that levels of ADAMTS-4 and its resulting ECM degradant ARGxx were higher in synovial fluid than those of ADAMTS-5 at the early stage of OA [38]. In this study of rat models, CZE significantly lowered ADAMTS-4 and ADAMTS-5 to normal levels dose dependently with a slightly stronger effect of CZE on ADAMTS-4 than on ADAMTS-5. This suggests that CZE can be used from the beginning stage of OA.

SOX9 acts as a transcription factor for ECM synthesis-related genes, such as *COL2A1* (a type 2 collagen gene), *ACAN* (an aggrecan gene) and *COMP* (a gene for the cartilage oligomeric matrix protein), in the early stage of OA progression. SOX9 is also regarded as an ADAMTS repressor by inhibiting its promoter gene. Inflammatory cytokines such as IL-1β and TNF-α inhibit the recruitment of SOX9, thereby enhancing the expressions of ADAMTSs in the early stage of OA progression [39]. The finding in this study that CZE restored SOX9 expression to near normal levels further supports the potential use of CZE from the beginning stage of OA progression, in line with the stronger effects of CZE on ADAMTS-4 than on ADAMTS-5. This is the first study to elucidate the relationship between SOX9 or ADAMTS and CZE. Furthermore, it is the first investigation to note that the marker compound of CZE, linarin, also has the same effects in OA progression and works by the same mechanism.

Indomethacin did not increase the synthesis of GAG in previous studies but rather damaged it [40]. In this study, indomethacin, a positive control, failed to increase SOX9, a transcription factor of GAG or *ACAN* genes and repressor of ADAMTS-4 and ADMATS-5.

## 5. Conclusions

Experiments using a rat model of OA and human chondrosarcoma cell line SW1353 revealed that CZE and its marker compound, linarin, can decrease the catabolism of articular cartilage via downregulation of ECM-degrading enzymes (MMPs and ADAMTSs) and increase the anabolism via upregulation of SOX9 and ECM synthesis-related genes transcribed by SOX9. Specifically, CZE can be used from the beginning stage of OA and it has a wider range of effects than previously known joint protectants. This study provided a clear mechanism for the use of CZE to prevent the progression of OA in humans.

## Figures and Tables

**Table 1:** Effect of CZE (100 mg/kg) on protein microarray analysis results in knee joints of MIA-induced OA rats.

Group 1: Pro-inflammatory Factors			Fold Change		
Gene Name	Full Protein Name	Antibody Name	C/N	S/N	S/C
Vcam1	Vascular cell adhesion molecule 1	CD106	1.657	1.380	0.833
Fadd	Fas-associated protein with death domain	FADD	1.579	1.190	0.753
Csf2	Granulocyte-macrophage colony-stimulating factor	GM-CSF	1.649	1.356	0.822
Ifng	Interferon-gamma	IFN-gamma	1.305	1.135	0.870
Il1a	Interleukin-1 alpha	IL-1 alpha	1.261	1.047	0.830
Il1b	Interleukin-1 beta	IL-1 beta	1.568	1.073	0.684
Il3	Interleukin-3	IL-3	1.253	1.205	0.962
Il4	Interleukin-4	IL-4	1.661	1.299	0.782
Il5	Interleukin-5	IL-5	1.453	1.112	0.765
Il10	Interleukin-10	IL-10	1.553	1.195	0.769
Il13	Interleukin-13	IL-13	1.779	1.299	0.730
Cxcl10	Interferon gamma-induced protein 10	IP-10	1.572	1.141	0.726
Ccl2	Monocyte chemoattractant protein 1	MCP-1	1.744	1.292	0.740
Mif	Macrophage migration inhibitory factor	MIF	1.337	1.060	0.793
Ccl3	Macrophage inflammatory protein 1-alpha	MIP-1 alpha	1.295	1.327	1.025
Cxcl2	Macrophage inflammatory protein 2-alpha	MIP-2 alpha	1.363	1.091	0.800
Ccl20	Macrophage inflammatory protein 3-alpha	MIP-3 alpha	1.362	1.044	0.766
Tgfb1	Transforming growth factor beta 1	TGF-beta1	1.216	0.960	0.789
Tgfb3	Transforming growth factor beta 3	TGF-beta3	1.039	0.735	0.707
Tnf	Tumor necrosis factor alpha	TNF-alpha	1.482	1.164	0.785
Tnfsf10	TNF-related apoptosis-inducing ligand	TRAIL	1.531	1.240	0.810
Tnfrsf19	TNF receptor superfamily, member 19	TROY	1.443	1.470	1.019
**Group 2: MMPs**			**Fold Change**		
**Gene Name**	**N**	**Antibody Name**	**C/N**	**S/N**	**S/C**
Mmp2	Matrix metalloproteinase-2 (gelatinase A)	MMP-2	1.202	1.269	1.056
Mmp8	Matrix metalloproteinase-8	MMP-8	1.071	0.735	0.686
Mmp13	Matrix metalloproteinase-13	MMP-13	1.409	0.732	0.520
**Group 3: Other Factors**			**Fold Change**		
**Gene Name**	**Protein Full Name**	**Antibody Name**	**C/N**	**S/N**	**S/C**
Egfr	Epidermal growth factor receptor	EGFR	1.521	1.128	0.741
Nrp2	Neuropilin 2	Neuropilin-2	1.868	1.022	0.547
Prlr	Prolactin receptor	Prolactin R	1.276	0.994	0.779
Vegfa	Vascular endothelial growth factor	VEGF	1.506	1.183	0.786
Adipoq		Adiponectin	0.934	1.788	1.914
Ide	Insulin degrading enzyme	IDE	0.937	1.456	1.554
Retnlg	Resistin-like molecule	RELM beta	0.695	1.088	1.566
Retn		Resistin	1.262	0.928	0.736
Ubiquitin		Ubiquitin	0.697	1.854	2.659

N, normal; C, MIA-induced control; S, CZE (100 mg/kg) sample after MIA induction. MIA, monosodium iodoacetate; CZE, *Chrysanthemum zawadskii var. latilobum*; OA, osteoarthritis; TNF, Tumor Necrosis Factor.
